# Sixty years since the creation of Lake Kariba: Thermal and oxygen dynamics in the riverine and lacustrine sub-basins

**DOI:** 10.1371/journal.pone.0224679

**Published:** 2019-11-05

**Authors:** Elisa Calamita, Martin Schmid, Manuel Kunz, Mzime Regina Ndebele-Murisa, Christopher H. D. Magadza, Imasiku Nyambe, Bernhard Wehrli

**Affiliations:** 1 Institute of Biogeochemistry and Pollutant Dynamics, ETH Zürich, Zürich, Switzerland; 2 Eawag, Surface Waters—Research and Management, Swiss Federal Institute of Aquatic Science and Technology, Kastanienbaum, Switzerland; 3 Department of Freshwater and Fishery Sciences, Chinhoyi University of Technology, Chinhoyi, Zimbabwe; 4 Department of Biological Sciences, University of Zimbabwe, Harare, Zimbabwe; 5 School of Mines, UNZA, Lusaka, Zambia; Universidad Federal de Minas Gerais, BRAZIL

## Abstract

The current boom of dam construction at low latitudes endangers the integrity and function of major tropical river systems. A deeper understanding of the physical and chemical functioning of tropical reservoirs is essential to mitigate dam-related impacts. However, the development of predictive tools is hampered by a lack of consistent data on physical mixing and biogeochemistry of tropical reservoirs. In this study, we focus on Lake Kariba (Southern Africa), the largest artificial lake in the world by volume. Kariba Dam forms a transboundary reservoir between Zambia and Zimbabwe, and therefore its management represents a socio-politically sensitive issue because the Kariba Dam operation completely changed the downstream hydrological regime. Although Lake Kariba represents a unique and scientifically interesting case study, there is no consistent dataset documenting its physical and chemical behaviour over time. This limits the scope for quantitative studies of this reservoir and its downstream impacts. To address this research gap, we aggregated a consistent database of in situ measurements of temperature and oxygen depth profiles for the entire 60 years of Lake Kariba’s lifetime and performed a detailed statistical analysis of the thermal and oxygen regime of the artificial lake to classify the different behaviours of the lake’s sub-basins. We demonstrate that the seasonal stratification strongly depends on the depth of the water column and on the distance from the lake inflow. Satellite data confirm these spatiotemporal variations in surface temperature, and reveal a consistent longitudinal warming trend of the lake surface water temperature of about 1.5°C from the inflow to the dam. Finally, our results suggest that the stratification dynamics of the lacustrine sub-basins have the potential to alter the downstream Zambezi water quality. Future research should focus on assessing such alterations and developing strategies to mitigate them.

## Introduction

Tropical ecosystems are nowadays experiencing the largest transformative changes [1,2]. Almost 40% of the world's population live in tropical areas, and this population is projected to increase by 50% by the end of 2050 [3]. The African continent has the highest population growth rate, and it is experiencing major anthropogenic changes of its landscapes which are driving diverse ecohydrological alterations [4,5]. Among others, rivers at low latitudes are experiencing a boom in hydropower dam construction [6]. Such dam construction causes alterations in natural water quantity and quality. The increased hydrologic residence time imposed by dams and the potential for stratification of their reservoirs can lead to major water quality alterations and particularly to shifts in the temperature and oxygen content of outflowing water [7–11].

Temperature and oxygen are fundamental to the functioning of aquatic ecosystems. The thermal regime plays a crucial role in triggering the life cycles of aquatic organisms such as the timing and duration of egg incubation, influencing species distribution patterns and competition [12,13]. Oxygen dynamics also directly impact aquatic ecosystems, influencing biogeochemical processes. In particular, hypoxia can cause the collapse of oxic river ecosystems, leading to potential extirpation of fish and other fauna [14]. Artificial alterations of temperature and oxygen dynamics can have cascading effects on other water quality parameters, on the integrity of aquatic ecosystems, and on associated services [15].

In order to address how dams affect the thermal regime and the oxygen concentration of the downstream river system we need to understand the internal thermal and chemical stratification dynamics of artificial reservoirs. The chemical and stratification dynamics are often spatially heterogeneous in reservoirs because of their dendritic shape resulting from the filling of a river basin. Indeed, longitudinal gradients between inlets and outlets are often more pronounced in reservoirs compared to natural lakes [16]. Such differences in physicochemical processes have cascading effects on the ecosystem. As a consequence, it is important to characterize stratification dynamics and possible heterogeneities in reservoirs. Moreover, it is necessary to clearly identify the internal functioning of the portion of reservoir where water is withdrawn, in order to analyse the dam’s impacts on the downstream river system.

The characterization of reservoirs relies on water quality databases, which provide in situ measurements of reservoir water properties over time. However, no comprehensive database exists detailing the water quality of tropical reservoirs. This research gap is well recognized by the scientific community, and several authors outlined its negative impact for further research [2,17–20]. Many studies on the effects of climate change on lake stratification and oxygen regimes had to rely on limited data, which led to difficult interpretations of results and often divergent conclusions [18,21–24]. To overcome this problem and to stimulate further research, the available knowledge on tropical reservoirs must be synthesized in order to initiate continuous and consistent water quality databases. Moreover, databases of in situ measurements of water properties are prerequisites for the development of modelling tools which could inform the future management of such waterbodies. Such data-driven modelling tools can integrate science and engineering communities with local stakeholders, governmental planners and industry, thus triggering a multidisciplinary context, which is at the base for a more sustainable management of water resources [5].

On the African continent, the Zambezi River is one of the most dammed rivers, and more than 15 new dams are planned in this river basin [25]. Among others, the Zambezi River Basin hosts Lake Kariba, the oldest artificial lake in the basin and the largest artificial reservoir in the world by volume. Lake Kariba was created in 1958 by damming the Zambezi River water at the border between Zambia and Zimbabwe. This transboundary reservoir was created for hydropower purpose and it has a socio-economic value not only for the Zambezi River Basin but for all south-eastern Africa [26]. The installation of the first generation units on the south bank of Lake Kariba was completed in late 1959, and the first electricity was generated in January 1960 [27]. Interest in the newly created lentic ecosystem in the Zambezi River Basin appeared only several years later when the scientific community started to consider environmental concerns such as the alteration of downstream water quality. Despite the lack of continuity, some information about the early development of the reservoir is available from these first studies. Now, 60 years after its creation, the lake also has an important role in the food chain of the river basin, providing fish for both local and regional consumption. Its economic value is higher if we also consider the touristic attraction that it represents in the Zambezi River Basin [17]. Because of its large superficial area and volume, Lake Kariba also influences the local rainfall regime, where one of the possible causes is the formation of a lake breeze system [28].

In this study, we aggregated a database of water temperature and dissolved oxygen profiles for the 60 years lifetime of Lake Kariba for the two following objectives: (i) to determine and quantify the spatial heterogeneity of thermal dynamics across this large artificial reservoir by using in situ measurements and satellite data and; (ii) to generate a comprehensive assessment of the thermal and oxygen regime in the riverine and lacustrine sub-basins of Lake Kariba. To our knowledge, this is the first study to look at Lake Kariba’s water thermal and oxygen dynamics by using a long-term dataset. Thus, our results can be used as a basis for forming further hypotheses about spatial patterns in biology or responses to climatic changes as well as for informing local or transboundary policies and management actions.

## Methods

### Environmental description of Lake Kariba

**Fig 1:**
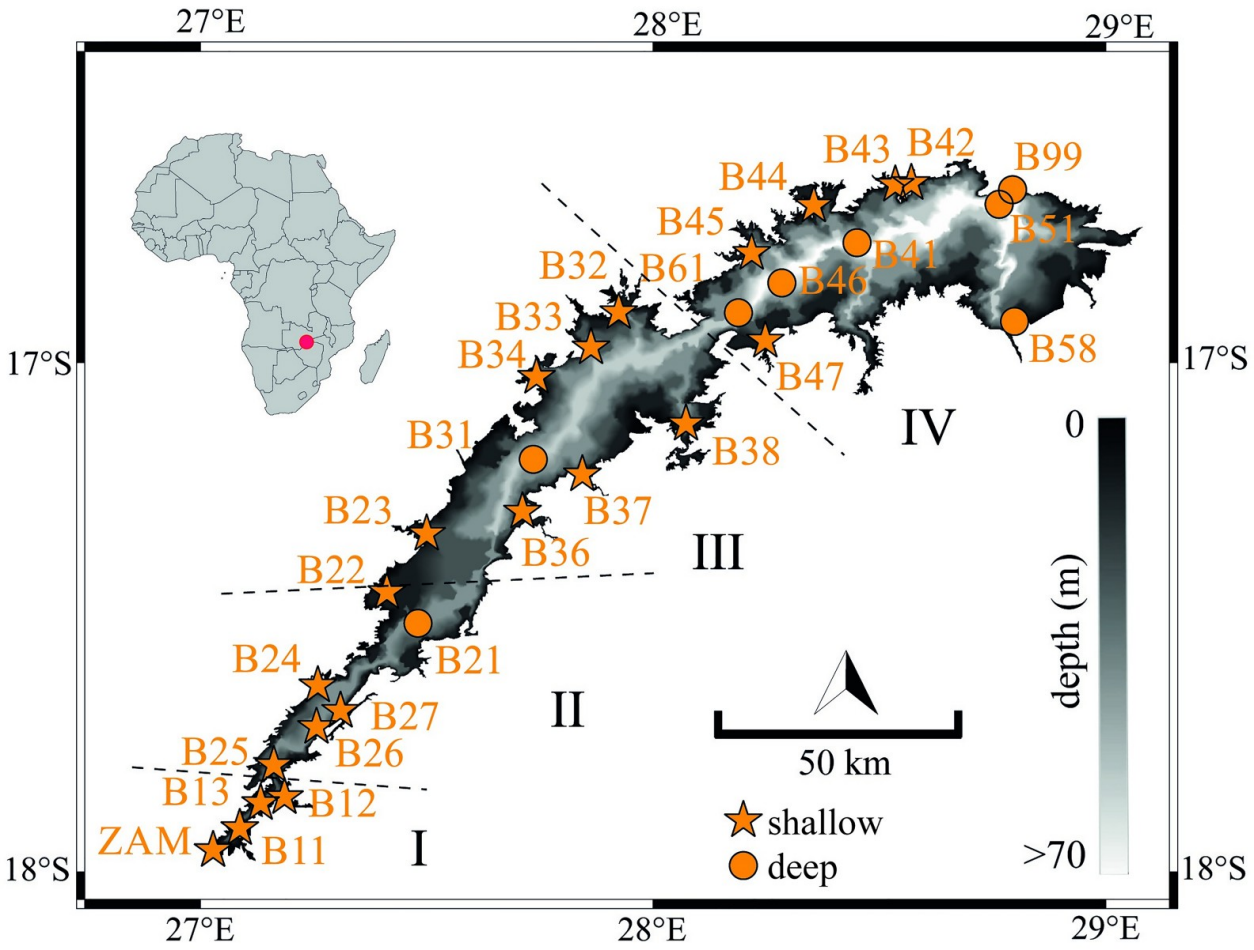
Geographical location in the Zambezi River Basin (Southern Africa) and bathymetric map of Lake Kariba. Orange markers indicate locations of in situ vertical profiles of temperature and dissolved oxygen at sites deeper (circles) or shallower (stars) than 50 m. Dashed lines separate the four sub-basins of Lake Kariba, and the naming of the sites corresponds to the open data collection of this study [34].

With a capacity of 180 km^3^, Lake Kariba is the biggest artificial lake in the world by volume [29]. It has a surface area of 5400 km^2^ and a maximum depth of 97 m. Lake Kariba is the major artificial reservoir along the Zambezi River Basin. It is located between 17.97° S and 16.45° S and stretches across two longitudinal degrees, from 27.00° E to 29.05° E (Fig 1). Lake Kariba lies at the border between Zambia and Zimbabwe, and it is shared in almost equal proportions by the two countries [30].

Lake Kariba and its catchment lie in the subtropical region. This geographic area experiences a pronounced wet season during the passage of the Intertropical Convergence Zone (November–March). The rest of the year the region is influenced by high pressure, and dry and sunny conditions prevail [31,32]. The Zambezi River is the main inflow of Lake Kariba and contributes on average 80% of the total lake inflow [31]. The remaining 20% are supplied by tributaries, among which the Sanyati River is the largest, and by direct rainfall on the lake surface. The average discharge of the Zambezi River at the inflow is about 7 times as large during the wet (~ 3500 m^3^ s^-1^) than during the dry (~ 500 m^3^ s^-1^) season (e.g. [33]).

Lake Kariba has an elongated and dendritic shape. Its longitudinal axis follows a SW-NE direction and its depth increases from the inflow toward the dam (Fig 1). For topographic reasons, Lake Kariba has been described as divided in four or five arbitrary sub-basins. In this study we adopted the division in four sub-basins (Fig 1) as presented by Balon and Coche [31]. The four sub-basins are separated by narrower lake zones and/or a series of islands. The first and second sub-basins are shallower and smaller in terms of surface area and volume in comparison to the other two sub-basins. The third and fourth sub-basins are deeper and contain 90% of the total reservoir capacity. The properties of the four sub-basins are reported in Table 1.

**Table 1:** Properties of the four sub-basins of Lake Kariba. List of main properties of the four sub-basins and the entire lake for the lake level of 485 m a.s.l. [26,31].

	Sub-basin I	Sub-basin II	Sub-basin III	Sub-basin IV	Entire Lake
Name	Mlibizi	Binga	Sengwa	Sanyati	Kariba
Max depth (m)	37.0	52.0	66.0	97.0	97.0
Mean depth (m)	12.6	24.0	26.5	33.2	29.2
Length (km)	23.0	56.0	96.0	102.0	277.0
Area (km^2^)	91.0	677.0	2033.0	2563.0	5364.0
Volume (km^3^)	1.1	16.3	54.0	85.1	156.5

### Database aggregation

To assess the spatio-temporal heterogeneity of Lake Kariba’s water, an aggregation of vertical profiles of water temperature (in°C) and dissolved oxygen (in mg L^-1^) from different sources has been completed. For the spatial analysis of the stratification dynamics we aimed at collecting vertical profiles well distributed all over the lake area and measured during the same period. To explore the stratification dynamics of Lake Kariba we focused on sub-basin IV of Lake Kariba where the lake is deeper and, as we will explore later, the influence of the inflow does not affect its stratification dynamics. We collected vertical profiles measured at similar locations in sub-basin IV and covering as many years as possible out of the 60 years of Lake Kariba’s lifetime.

#### Spatial database

We assembled spatially distributed thermal and oxygen profiles for the period 2007–2009. These profiles were measured during the doctoral project of Manuel Kunz (Eawag) using a temperature-oxygen-depth probe (CTD; CTD60M, Sea and Sun Technologies), and oxygen concentrations were cross validated with Winkler measurements. The profiles are well distributed in space over the lake and cover all four sub-basins of Lake Kariba (Fig 1). Moreover, they cover both shallow and deep portions of the lake. Profiles were measured down to the local lake bottom. Coordinates and sampling dates are provided together with the database [34].

#### Temporal database

We collected the published records and added previously unpublished profiles measured by Eawag (Switzerland) and by the Environmental Monitoring Programme of the Zambezi River Authority (ZRA) as well as our own measurements carried out during this study. All profiles have been measured in sub-basin IV of Lake Kariba. Sources of all data included in the database are listed in Table 2.

**Table 2:** Data sources. Overview of the data sources of observed vertical profiles of water temperature and dissolved oxygen included in this study.

*Years*	*Institute*	*Reference*
*1964–1965*	Fishery Limnologist, FAO Central Fisheries Research Institute, Zambia	Coche, 1968 [35]
*1988–1992*	University Lake Kariba Research Station, Zimbabwe	Magadza *et al*., 1987 [36]; Magadza *et al*., 1988 [37]; Magadza, 2010 [26]
*2002–2018*	Zambezi River Authority, Zambia	-
*2007–2009*	University of the Western Cape, Republic of South Africa	Ndebele-Murisa *et al*., 2014 [22]
*2007–2009*	Eawag, Switzerland	-
*2018*	Eawag, Switzerland	This study

The methods and instruments used for measurements were different for each data source, and as a result, the depth resolutions of the profiles are not consistent. Methods and resolutions of published profiles are described in the cited references; for the other data, we report the sampling regime and methods. Profiles measured by Eawag between 2007 and 2009 were recorded with the same method described for the spatially distributed profiles presented in the previous section. These profiles represent the first continuous measurements along the depth of Lake Kariba’s water column in our database. All previous measurements, as well as those measured by the Zambezi River Authority in their Environmental Monitoring Programme, were taken at discrete depths. Since 2007, the Zambezi River Authority has measured monthly water temperature and dissolved oxygen concentration in sub-basin IV (location B51 of Fig 1) of Lake Kariba at five depths (0.5, 10, 20, 30 and 50 m), where the maximum water depth is approximately 90 m. Finally, the latest two profiles were recorded in March and July 2018 using a temperature-oxygen-depth probe (EXO2 probe, YSI), thus, they are continuous measurements along the water column.

We obtained 236 lake profiles for the sub-basin IV of Lake Kariba. Observations cover 23 years with the oldest records from 1964. The proposed database exhibits some gaps, however, since 2007 it offers a continuous record with a monthly time resolution. All in situ measurements used in this study are freely available at [34].

### Satellite data

The spatial database was coupled with satellite data to better understand the spatial variability of surface water temperature in Lake Kariba. We used high resolution images from the NASA Group for High Resolution Sea Surface Temperature (GHRSST) Level 4 Multi-scale Ultrahigh Resolution (MUR) to obtain the lake surface temperature. The satellite data is freely available through the data portal on the NASA website (https://podaac.jpl.nasa.gov/dataset/MUR-JPL-L4-GLOB-v4.1). Lake surface temperature data are produced by merging data from different satellites and sensors (IR and Microwave) and in situ measurements [38]. This GHRSST database has been validated, and error estimates are provided for different components such as sensor errors and atmospheric conditions [39]. The Multi-scale Ultrahigh Resolution (MUR) analysis we used is based on night-time skin and sub-skin lake surface temperature observations from NOAA’s sensors, and Aqua and Terra satellites which include: the NASA Advanced Microwave Scanning Radiometer-EOS (AMSRE), the Moderate Resolution Imaging Spectroradiometer (MODIS), microwave WindSat radiometer, Advanced Very High Resolution Radiometer (AVHRR) as well as in situ observations from NOAA iQuam research. For this project, we used data for Lake Kariba from 2007 to 2009 with spatial resolution of 0.01 x 0.01 latitudinal and longitudinal degrees (~1.25 x 1.25 km) and daily temporal resolution. Satellite-retrieved images have been recognized as a useful tool to assess the heterogeneity of freshwater skin temperature [40–42]. In our case, the high spatial resolution together with the daily time resolution of the retrieved images enabled a detailed analysis of the thermal dynamics of the lake.

### Data analysis

#### Spatial analysis

We performed a spatial analysis of the temperature and oxygen lake profiles in order to determine the spatial and temporal heterogeneity of Lake Kariba's thermal and oxygen dynamics. Moreover, such analysis allowed us to quantify the differences of the lake thermal and oxygen regime in each sub-basin over different seasons. For this analysis, we considered the spatially distributed profiles from 2007–2009 and the satellite data during the same time window. In order to distinguish between shallow and deep parts of the lake we classified each lake location as deep or shallow according to its maximum depth with a threshold of 50 m. We analysed the vertical profiles measured during February (warm-wet season) and during July (cold-dry season).

The spatial analysis is summarised in the following steps. First, we clustered the vertical temperature and oxygen profiles into four classes based on their sub-basins, and we divided them into two groups based on the season (warm-wet and cold-dry). Second, we analysed the strength of thermal and chemical stratification in the warm-wet and cold-dry season for all four sub-basins of Lake Kariba. We used the water temperature difference between the upper- and the lowermost 5 m layer (Δ temperature in°C) as an index of thermal stratification. Our chemical stratification index is based on the chemical stratification strength proposed by Yu *et al*. in 2010 [43]. Chemical stratification strength for dissolved oxygen (IC-DO) is defined as the ratio of the dissolved oxygen concentration difference between epilimnion and hypolimnion to the average concentration along the water column. The epilimnion and hypolimnion concentrations were calculated as the mean of the top and bottom five meters of the recorded profiles, respectively. Finally, we statistically tested the null hypothesis of equal means for the thermal stratification strength of shallow and deep locations during both the warm-wet and the cold-dry season, and we calculated the confidence intervals for the thermal stratification strength of the different identified groups.

Residence time was calculated for each lake sub-basin as the ratio between the water volume of the sub-basin and the average flow of the Zambezi River of 1200 m^3^ s^-1^ [44]. Moreover, we calculated the densimetric Froude number (*Fr*), which is useful for characterizing a lake or reservoir in terms of its tendency to stratify [45]. This index compares the internal force, represented by the average flow-through velocity, with the gravitational force required to maintain stability [45]. The densimetric Froude number is considered as an average normalized density gradient in the reservoir [46], and it reads *Fr* = 320*(L*Q)/(D*V). The coefficient 320 has the dimension of time, L is the length of each sub-basin (in m), Q is the average inflowing water (in m^3^ s^-1^), D is the average depth (in m) and V is the volume of each sub-basin (in m^3^) at 485 m lake level [47,48]. Particularly, the densimetric Froude number is always positive and its value is an indicator of the tendency of the reservoir to stratify. Typical values are 0<*Fr*<1/*π* for stratified reservoirs, 1/*π*<*Fr*<1.0 for weak stratification, and *Fr*>1.0 for absence of stratification.

The surface water temperature variability from in situ measurements was compared with satellite data in order to better assess the longitudinal zonation of Lake Kariba. We calculated the mean February and July water temperature maps in the years 2007–2009 by averaging the daily values over the same period of 2007–2009. Then, we calculated the monthly spatial water temperature anomalies of surface water temperature for each lake cell as the difference between the mean temperature value (*T*_*m*,*i*_) in that specific cell with the averaged surface temperature in that month (*T*_*m*_):

$$T_{m,i}^{\prime }=T_{m,i}-\bar{T_{m}}$$ (1)

The apostroph′ indicates anomalies, the subscript *m* denotes the *m*-th month of the year, and *i* represents the cell of the lake. The use of temperature anomalies is a common and consolidated practice to describe intra-lake variability in climate change studies [49,50].

#### Statistical analysis

In order to generate a comprehensive assessment of the thermal and oxygen regime in the lacustrine sub-basins of Lake Kariba we performed a statistical analysis of the thermal and oxygen historical data of sub-basin IV. Particularly, this analysis aims at (i) statistically explaining the characteristics of stratification in Lake Kariba, (ii) characterizing the behaviour of water temperature and dissolved oxygen at different depths in the first 50 meters of the lake water column, (iii) studying the correlation between thermal and oxygen dynamics in the lake, and (iv) quantifying the areal hypolimnetic oxygen demand in sub-basin IV of Lake Kariba.

We analysed the aggregated database of vertical profiles for water temperature and dissolved oxygen by considering five different depths: 1 m, 10 m, 20 m, 30 m and 50 m. For each selected depth d_i_ we built two time series, one for water temperature and the other for dissolved oxygen, by averaging for each profile the observed measurements in the range *d*_*i*_±5 *m*. First, we analysed the range of variation of the two water properties at the five depths. Second, we calculated the empirical cumulative distribution function of water temperature and dissolved oxygen at each depth (S1 Fig). Finally, from the empirical cumulative distributions we derived depth-temperature and depth-oxygen frequency maps. The probability was proportionally converted in days per year to have a quantification in days of the relative temperature-depth and oxygen-depth conditions. We repeated the statistical analysis for each month of the year. The monthly hypolimnetic oxygen concentration allowed us to calculate the areal hypolimnetic oxygen depletion rate which represents the rate of change of the hypolimnetic oxygen deficit (AHOD, g m^-2^ d^-1^) [51]. Thus, the oxygen depletion rate (AHOD) is the rate of loss of the mass of DO, normalized for the surface area of the hypolimnion [52].

## Results and discussion

### Time series assembly

**Fig 2:**
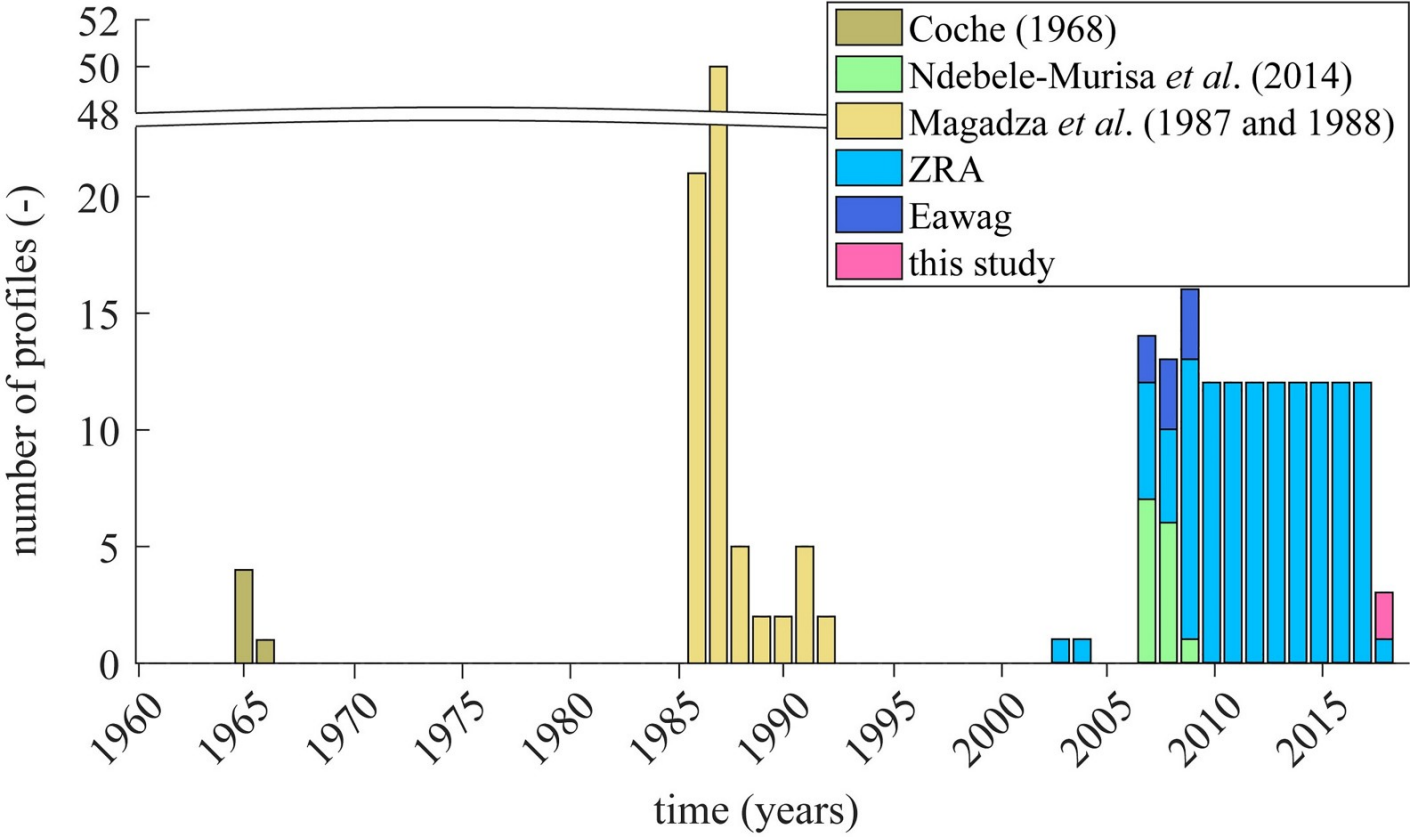
Aggregated database of Lake Kariba’s vertical profiles. Coloured areas represent the years when measured profiles of lake water temperature and dissolved oxygen are available. The legend reports data sources for the lake vertical profiles where ZRA stands for Zambezi River Authority.

The aggregated database for Lake Kariba includes 273 vertical profiles of both, water temperature and dissolved oxygen. Among these, 37 profiles are well distributed all over the lake surface (Fig 1). The rest of the database contains 236 lake profiles distributed over sub-basin IV covering 23 years in the period 1964–2018. Fig 2 shows the time distribution of the thermal and oxygen profiles, and their sources are listed in the legend. The lake profiles are irregularly distributed over time.

The time distribution of profiles in our database highlights how in recent years the monitoring of Lake Kariba has been more consistent and continuous (Fig 2). Despite the cost and effort to carry this kind of water monitoring programme, local authorities and decision makers recognise the importance of such a monitoring programme to better understand the functioning of freshwater ecosystems. This consciousness reflects the involvement of local stakeholders in interdisciplinary research projects that aim to link water management options with water quality responses and, more in general, environmental problems.

### Spatial heterogeneity

**Fig 3:**
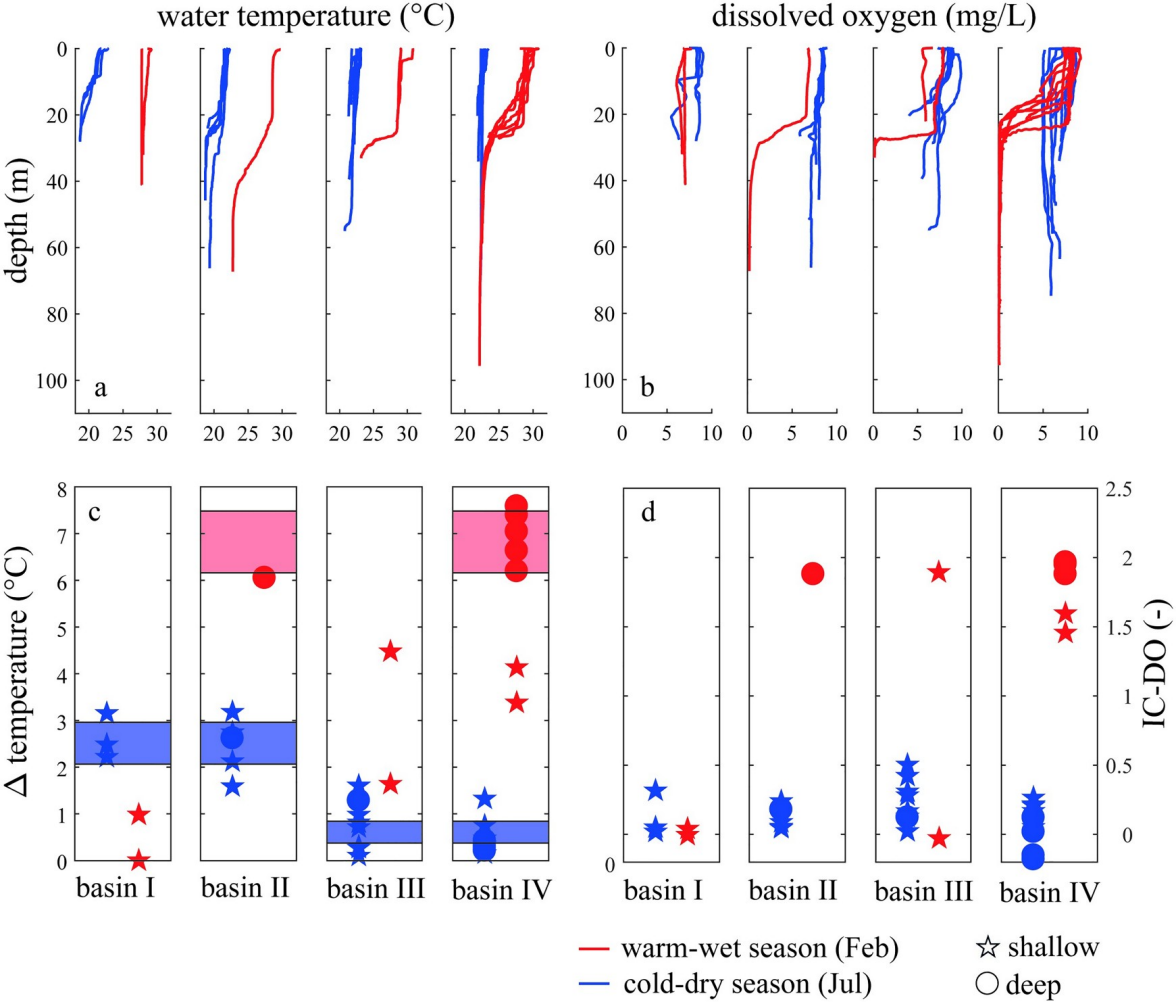
**Thermal and chemical stratification in the four sub-basins of Lake Kariba for February (red) and July (blue).** Observed vertical profiles of water temperature (a) and dissolved oxygen (b) in the four sub-basins of Lake Kariba. The strength of the stratification is shown as the temperature difference between the top and bottom 5 m in each profile (c). Strength of the oxygen stratification (d). Circles in panels c and d represent deep parts of the lake and stars represent shallow parts. The threshold between shallow and deep is 50 meters. Blue areas represent the 95% confidence intervals of the mean stratification strength during the cold-dry season in the first two and second two sub-basins, respectively. The red area represents the confidence intervals of the mean stratification strength during the warm-wet season for deep locations.

Our spatially distributed analysis of water temperature and dissolved oxygen across seasons confirms the longitudinal heterogeneity of Lake Kariba and underlines the different behaviour of sub-basins I and II in comparison to sub-basins III and IV. The first two sub-basins are classified as riverine because their stratification characteristics are largely driven by the dynamics of the inflow. Although usually the dynamics of lake stratification are mainly driven by meteorological forcing, inflows and outflows or groundwater can have a considerable role in lake stratification [53]. Density differences between inflow and lake water control the vertical distribution of inflowing river water into the reservoir. As a result, a river entering a lake can flow large distances as a gravity-driven density current. In our case study, during the cold-dry season the Zambezi River water temperature is colder than the lake water. This colder water has higher density, thus, the river water flows at the bottom of the water column. This is confirmed by the thermal stratification in the first two sub-basins during the cold-dry season, which is due to the river intrusion in the water column (Fig 3A). Since oxygen concentrations are close to equilibrium with the atmosphere both in the inflow and in the lake surface water, the oxygen profiles appear not stratified (Fig 3B). During the warm-wet season instead, sub-basin I does not present any stratification while sub-basin II shows a stratified profile. Sub-basin III and IV of Lake Kariba follow the stratification pattern of a tropical monomictic lake, thus, they are classified as the lacustrine sub-basins of Lake Kariba. The lake water column appears well mixed during the cold-dry season and the stratification occurs during the warm-wet season (Fig 3A). The oxygen profiles in these sub-basins follow the thermal stratification dynamics (Fig 3B).

Stratification strength has been further analysed by using two different indexes for thermal stratification and for chemical stratification (Fig 3C and 3D). This analysis quantifies the stratification differences between sub-basins and between shallow and deep zones of the lake. For both indexes, higher values correspond to stronger stratification, and shallow and deep zones of the lake are indicated with stars and circles, respectively. The thermal stratification indexes are not significantly different between shallow and deep zones during the cold-dry season (null hypothesis of equal means not rejected, p = 0.34), whereas the differences are evident during the warm-wet season (null hypothesis of equal means rejected, p = 2.38e-4). In particular, during the cold-dry season, the confidence interval for the mean stratification strength is higher in sub-basins I and II (2.06–2.96) than in sub-basins III and IV (0.38–0.84). Conversely, during the warm-wet season, the stratification strength at shallow locations (red stars in Fig 3C) increases from sub-basins I and II to sub-basins III and IV (Fig 3C, red stars below blue area in sub-basins I and II and above blue area in sub-basins III and IV). Thus, the stratification is stronger in the first two sub-basins during the cold-dry season, and in the second two sub-basins during the warm-wet season. Moreover, the deeper zones during the warm-wet season experience a strong stratification in every lake sub-basin (highest confidence interval, 6.16–7.48). Finally, the oxygen stratification strength depends more on the depth of the water column than on the sub-basin.

**Fig 4:**
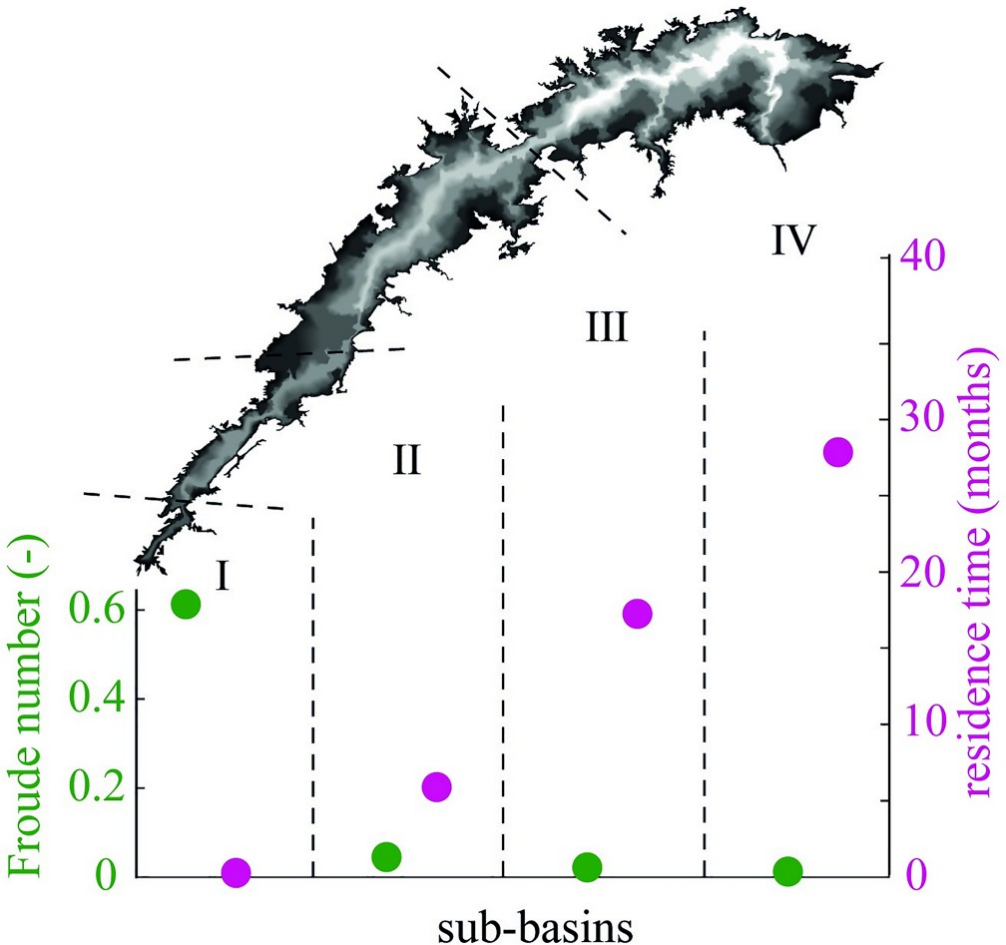
Froude number and water residence time. Froude number and water residence time calculated for each sub-basin of Lake Kariba. The Froude number decreases from the first toward the fourth sub-basin, and the water residence time increases toward the fourth sub-basin.

The profound differences between the first two and the second two sub-basins can be explained by the residence time of water in each sub-basin. Short residence times support a riverine behaviour while long residence times favour the build-up of thermal stratification. The water residence time increases from one week in sub-basin I to almost 2.5 years in sub-basin IV, and the Froude number (*Fr*) decreases along the same transect (Fig 4). The *Fr*<1/*π* in sub-basins II, III, IV indicate that they are more likely to stratify than sub-basin I. Water residence time plays an important role in the limnology of tropical manmade lakes [54,55]. Effects of different residence times can be seen not only among different lakes but also within the same waterbody and resulting in the typical spatial zonation [16]. By controlling the ratio of riverine and lacustrine portions in the lake, the water retention time controls most ecological processes, habitat availability, the productivity of the lake and as a result even the uptake or release of greenhouse gases. Previous studies demonstrated that the riverine portion contributes most to the atmospheric greenhouse gas emissions from Lake Kariba [56].

Lake Kariba’s zonation generates a longitudinal pattern in the lake water temperature. During February, the lacustrine sub-basins of Lake Kariba are stratified, and therefore the heat flux from the atmosphere is absorbed only by the well-mixed layer of the water column. By contrast, the riverine lake portion reflects the river water temperature. As a net effect, the surface water temperature of the lacustrine sub-basins is higher. During July instead, when the lacustrine sub-basins are well-mixed, the water temperature of the epilimnion approaches the temperature of the hypolimnion which is almost always constant at about 22°C. The higher thermal inertia during seasonal mixing of the lacustrine basins, where the entire water column participates in the heat exchange with the atmosphere, prevents the rapid temperature drop that is observed in the riverine sub-basins.

**Fig 5:**
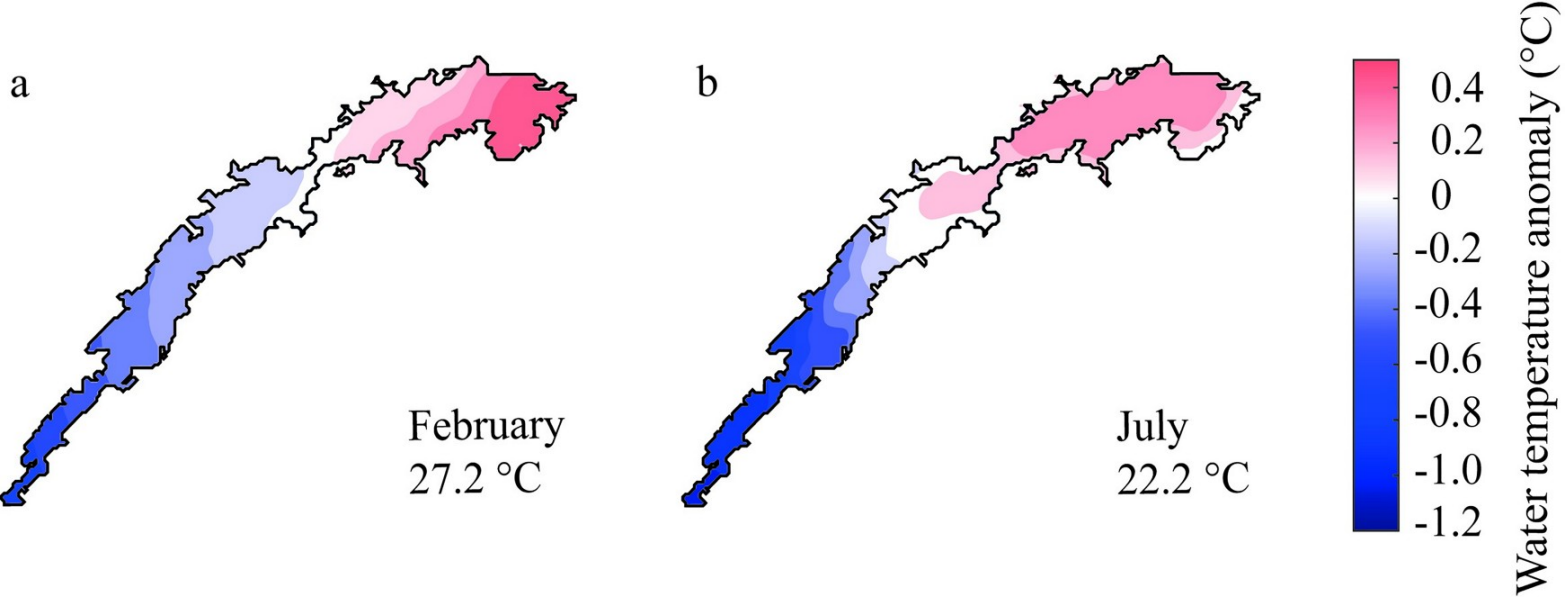
Anomaly maps of lake surface water temperature. Water temperature anomaly shown as spatial deviation from the satellite-derived mean monthly surface water temperature for the years 2007–2009 in (a) the warm-wet season (February) and (b) the cold-dry season (July).

Satellite data of surface water temperature capture this longitudinal trend, both during the warm-wet and the cold-dry season (Fig 5). Although in February the mean surface water temperature is on average about 5°C higher than in July, in both seasons the surface water temperature increases smoothly towards the dam with temperature differences of about 1.5°C between the last and the first sub-basin. The transition zone appears distributed over sub-basins III and IV in February, and more localized in sub-basin III in July. In fact, the extent of the riverine portion of the lake can change over the year. Lake zonation depends on the water residence time and this is strongly driven by the inflowing water regime and its strong seasonal variability [44]. This result agrees with Soares *et al*. [55] who showed that tropical reservoirs appear more spatially heterogeneous when the water residence time is longer (February). The same idea was later tested by Pacheco *et al*. [57], who demonstrated a longitudinal shift of the transition zone in tropical reservoirs between the dry and wet season.

**Fig 6:**
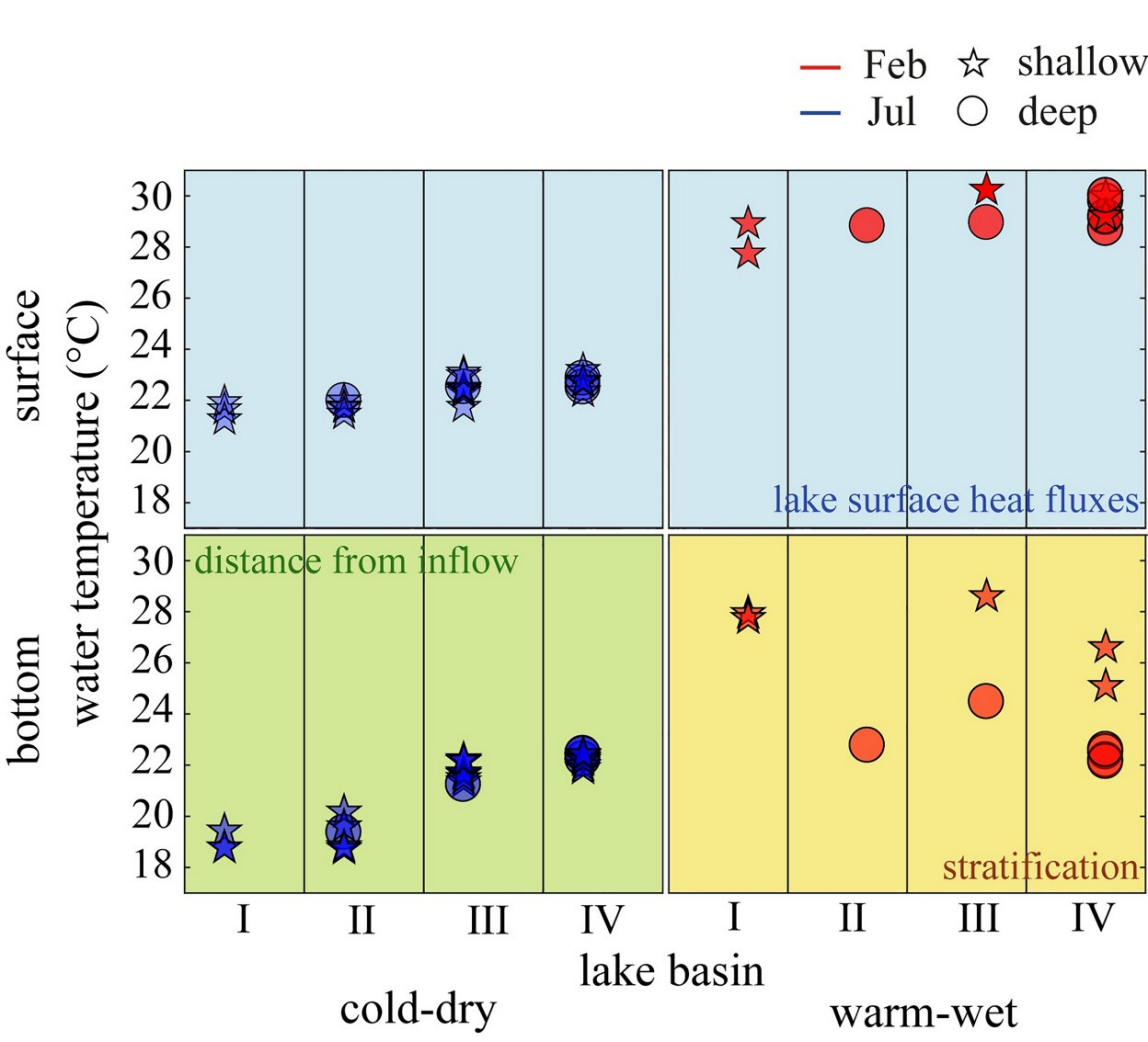
Spatio-temporal analysis of water temperature in Lake Kariba. Stars and circles represent locations where the lake is shallower and deeper than 50 m, respectively. Background colours highlight the different drivers of the lake water temperature.

In agreement with satellite data, in situ measurements of surface water temperature confirm the increasing trend from the first toward the last sub-basin during February and July (Fig 6). In all four sub-basins and in both months the surface water temperature does not vary with the local lake depth. On the contrary, the bottom water temperature shows different patterns between July and February, and during the latter month the shallow and deep zones behave differently. During July, the bottom-water temperature increases from the first toward the last sub-basin as the surface temperature, but the range of variation is about 3°C, twice that of the surface water temperature. In the riverine sub-basins, the deep water is colder than the surface water due to the deep intrusion of the colder Zambezi water. Conversely, the lacustrine sub-basins are well mixed, with homogenous temperature from top to bottom. During February, the water column in the lacustrine sub-basins is stratified, and temperature decreases with depth. Therefore, the bottom water temperature at a specific location depends mostly on the local depth.

Our analysis shows that not only the lake zonation but also the shift of the transition zone can be detected from satellite-retrieved images of surface water temperature. However, surface water reflects only in part the zonation of the reservoir. The main differences occur in the bottom water where the variability among lake sub-basins is greater. Deep water monitoring is therefore important for understanding the full dynamics in the lake system, both from the hydrodynamic and the chemical point of view.

Straskraba *et al*. [16] suggested that within-reservoir gradients in water quality and trophic status can be viewed as a positive attribute. They may create opportunities for optimizing reservoir management. These gradients indeed provide a diversity of potential water uses in a single water body. Moreover, the zonation of elongated deep-valley reservoirs favours the diversity of fish communities along their longitudinal axes [58]. The diversity in species and populations helps to maintain ecosystem services and promote resilience for future changes [59,60].

### Temporal analysis

**Fig 7:**
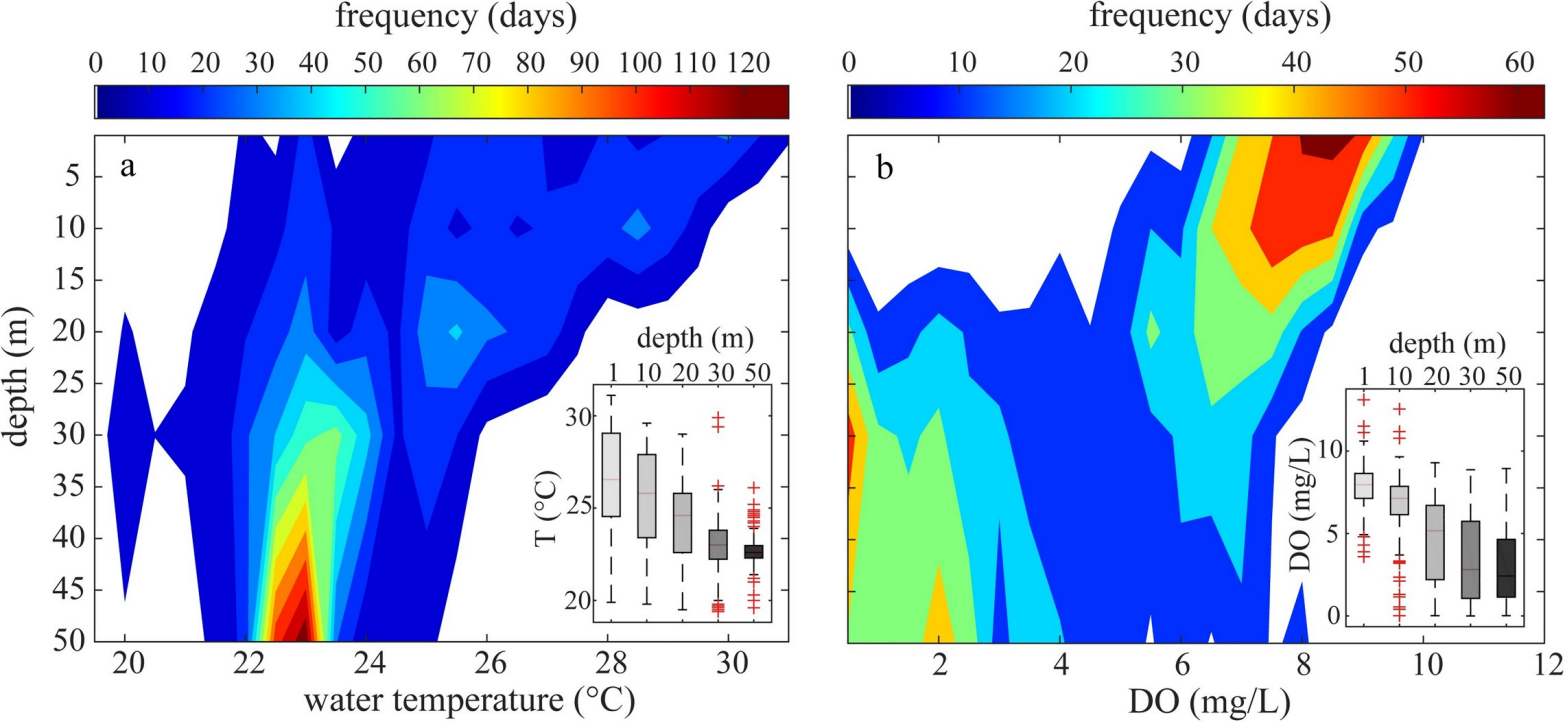
Frequency maps of lake water temperature and dissolved oxygen. Empirical frequency maps of (a) water temperature-depth and (b) oxygen as a function of water depth for Lake Kariba calculated by using the aggregated database of this study [34]. Frequency is quantified as the average number of days per year (colour-coded) in which observed temperatures and oxygen concentrations fall within a bin of 0.5°C for temperature and 0.5 mg L^-1^ for oxygen. The box plots show the statistical distributions (medians, 25^th^ and 75^th^ percentiles) of water temperature and dissolved oxygen at five different depths. The whiskers extend to the most extreme data points not considered outliers, and the outliers are plotted individually using the '+' symbol.

The stratified and well mixed conditions affect the water temperature and dissolved oxygen in the water column. To analyse how long different water temperature and dissolved oxygen conditions last at each depth in the water column, we built frequency maps (Fig 7). Such maps quantify the number of days per year during which a certain range of water temperatures and dissolved oxygen concentrations occur at a given depth in the water column of sub-basin IV. Moreover, in Fig 7, the overall variability of water temperature and dissolved oxygen is represented by the extents of the boxes (interquartile ranges) and of the whiskers for the five different lake depths. Median values of both water temperature and dissolved oxygen concentration show a decreasing pattern with depth in sub-basin IV of Lake Kariba.

The ranges of observed lake water temperatures in sub-basin IV are wider at the surface and narrower in the deep water. Kariba reservoir is an open system in direct contact with the atmosphere. Thus, the surface water tends to equilibrate with the atmosphere. Hence, its water temperature follows the seasonal variation of air temperature. The range of variation of water temperature decreases with depth because of the stratification dynamics occurring in sub-basin IV. Hypolimnetic water is isolated, and therefore its water temperature remains almost constant all over the year at about 22.5°C. Dissolved oxygen, conversely, increases its range of variation with increasing depth. The reason is again the stratification of the lake. The oxygen concentration in the epilimnion of Lake Kariba is always close to saturation. The resupply of DO to the hypolimnion through the thermocline is limited, which together with the oxygen consumption, generates anoxic conditions in the hypolimnion during the stratified season. During July, when mixing occurs in the water column, the oxygen in the hypolimnion tends to re-equilibrate.

The frequency maps in Fig 7 describe the frequency distribution of temperature and oxygen, which are defining the oxythermal habitats, at each lake depth over the year and quantify the duration of different conditions in terms of days per year. They also document the depth of the thermocline and oxycline. Specifically, around the depth of 25 m the probability distribution becomes bimodal, reflecting that two different conditions prevail at this depth. Indeed, the water temperature and even more the oxygen concentration show that at this depth there are two most probable conditions (e.g. *DO*<3 mg L^-1^ or *DO*>5 mg L^-1^). In a water management context, these maps represent a semi-quantitative tool to assess the water temperature and dissolved oxygen alterations occurring in the downstream river system. If water is withdrawn from a certain depth in the reservoir, the maps allow estimating the temperature and dissolved oxygen in the turbinated water. Given that the water intakes of Kariba Dam are located at depths ranging from 20 to 30 m, the temperature of the turbinated water ranges between 20 and 27°C, and its dissolved oxygen concentration varies seasonally between anoxia and saturation concentration.

**Fig 8:**
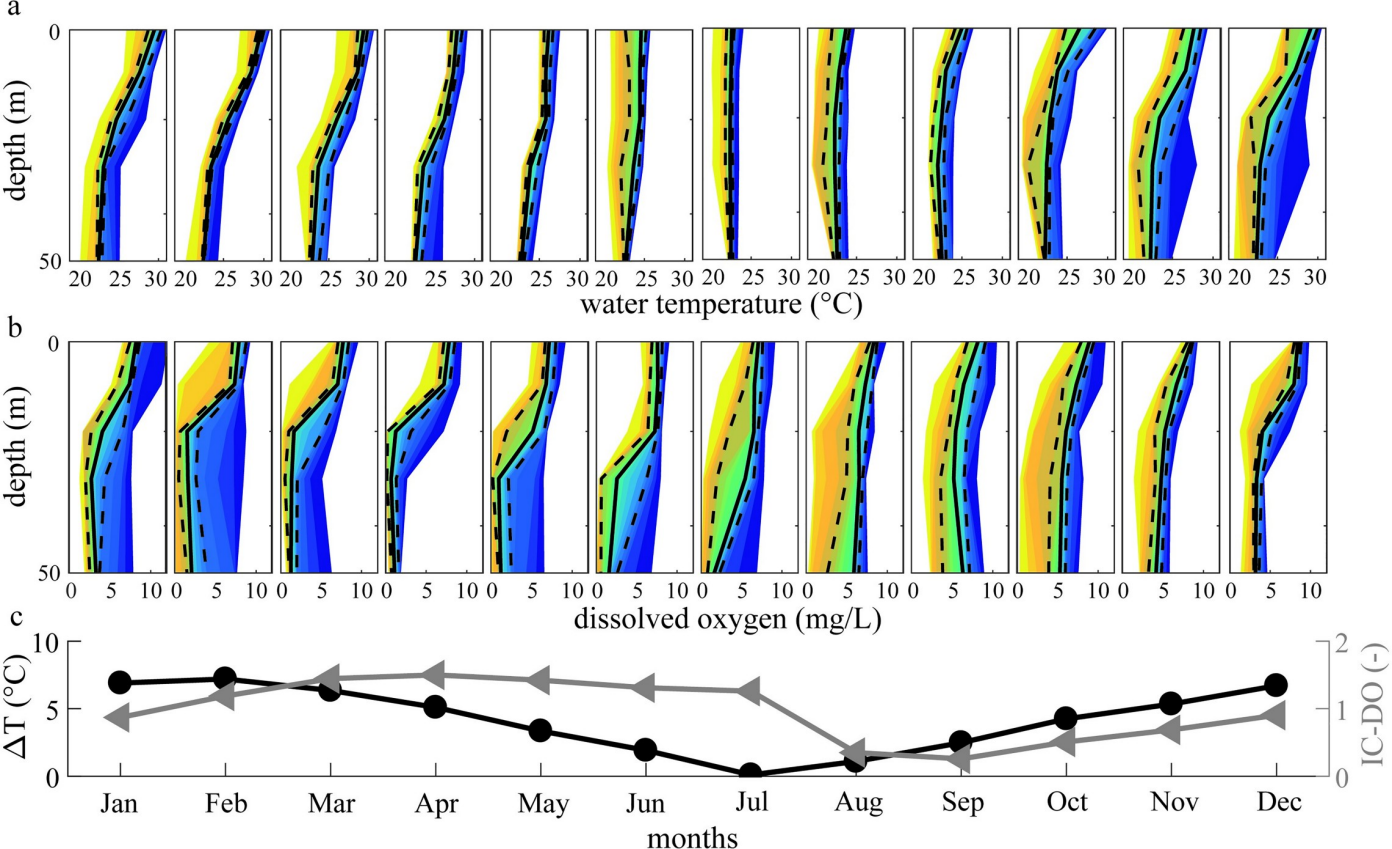
**Monthly** (a) **lake water temperature and** (b) **dissolved oxygen variability.** Solid black lines represent medians, dashed lines represent 25^th^ and 75^th^ percentiles, and the different colours represent the percentiles from 5^th^ to 95^th^ with a step of 5%. (c) Monthly stratification coefficients for water temperature (black dots) and dissolved oxygen (grey triangles) for each month of the year, using the temperature difference (°C) between the upper- and the lowermost 5 m layer of the median thermal profiles and the IC-DO of each month.

In order to assess when a certain condition occurs, we analyse the annual cycle of water temperature and dissolved oxygen in the water column (Fig 8). The monthly thermal stratification index shows that the thermal stratification reaches a maximum during February, and seasonal mixing usually occurs during July. The oxygen cycle follows the thermal dynamics with a delay of approximately 2 months (Fig 8C). Given that the flux of reduced substances from the sediment (e.g. methane) is one of the major factors consuming oxygen in the hypolimnion of lakes [61], and knowing that methane can accumulate to high concentrations during the stratified season in the hypolimnion of Lake Kariba [56], we can conclude that the delay of oxygen recovery during the mixing phase is likely due to the high amount of reduced substances stored in the hypolimnion which have to be oxidized before oxygen can be replenished.

**Fig 9:**
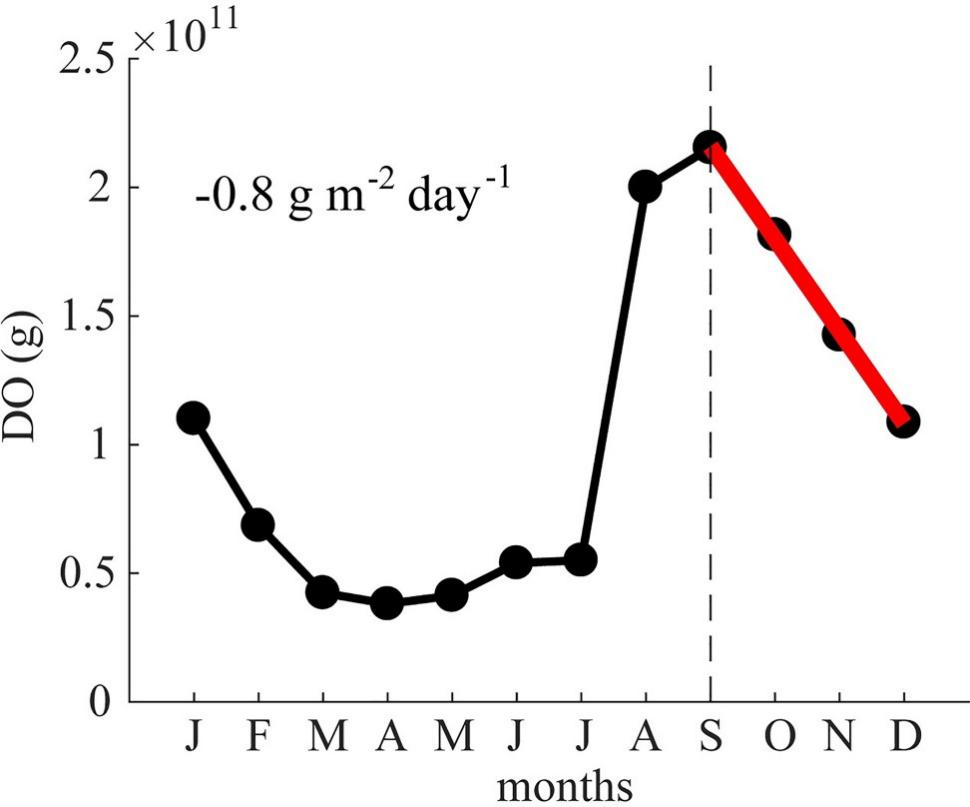
Hypolimnetic mass of dissolved oxygen in Lake Kariba. Mean year of dissolved oxygen mass in the hypolimnion of Lake Kariba. The mean year is based on the aggregated database of this study. The hypolimnion concentration was calculated as the mean of the deepest five meters. The red line represents the regression line used to calculate the areal hypolimnetic oxygen depletion rate (AHOD).

From the monthly median oxygen profiles, we estimated the AHOD, the rate of oxygen depletion per unit area of hypolimnion. Fig 9 shows the linear decrease of oxygen mass in the hypolimnion from September to December and the resulting AHOD of 0.8 g m^-2^ day^-1^. This value falls in the range of 0.40–0.83 g m^-2^ day^-1^ estimated by Balon & Coche [31] in 1974, suggesting that since then there was no major shift in the oxygen consumption. Lake Kariba’s AHOD is about double in comparison to the AHOD calculated for Itezhi-Tezhi Reservoir located in the same river basin [62]. Moreover, Kariba’s AHOD agrees quite well with a model fit of AHOD to mean hypolimnion depth observed in 11 eutrophic French and Swiss lakes [63]. The AHOD of Lake Kariba ranges in the middle of those observed in 30 Canadian and US lakes by Walker [51] (0.06–1.73 g m^-2^ day^-1^), and according to the classification proposed in the same study, Lake Kariba should be an eutrophic lake. However, this classification has been developed for temperate lakes, so its application to tropical lakes can be misleading.

Hypolimnetic oxygen depletion reflects decomposition of settling and deposited particulate organic matter formed by primary producers in the upper trophogenic zone or transported into the lake from terrestrial sources. In tropical lakes, the primary production is usually high (even if nutrient concentrations are quite low) and therefore they are particularly prone to loss of deep-water oxygen [64–67]. Moreover, given the long stratification season in tropical lakes, the deeper waters of tropical lakes are predominantly anoxic, whereas those of temperate lakes may be either anoxic or oxic, depending largely on the trophic state and mean depth of the lake [68]. Thus, the classification of the trophic state based on nutrient concentrations in the epilimnion is not necessarily a good indicator for the occurrence of anoxic conditions in the hypolimnion of tropical lakes. Lake Kariba, indeed, has always been described in literature as oligotrophic in terms of nutrients concentration but its deeper water experiences anoxia [31,56].

Nowadays, climate change is imposing a warmer climate even in tropical regions. Among other effects, the predicted air temperature increase would increase lake water temperature, thus the bacterial metabolism will increase, resulting in more organic carbon respiration [69]. Together with stronger stratification, climatic warming will increase the risk of occurrence of deep-water anoxia. Thus, it is crucial to know the lake oxygen consumption in tropical lakes to facilitate sustainable use of lake water and conservation of endemic species [70]. Due to poor representation in global datasets, describing the baseline functioning of tropical lakes and reservoirs is particularly important.

### Thermal-oxygen cycle

**Fig 10:**
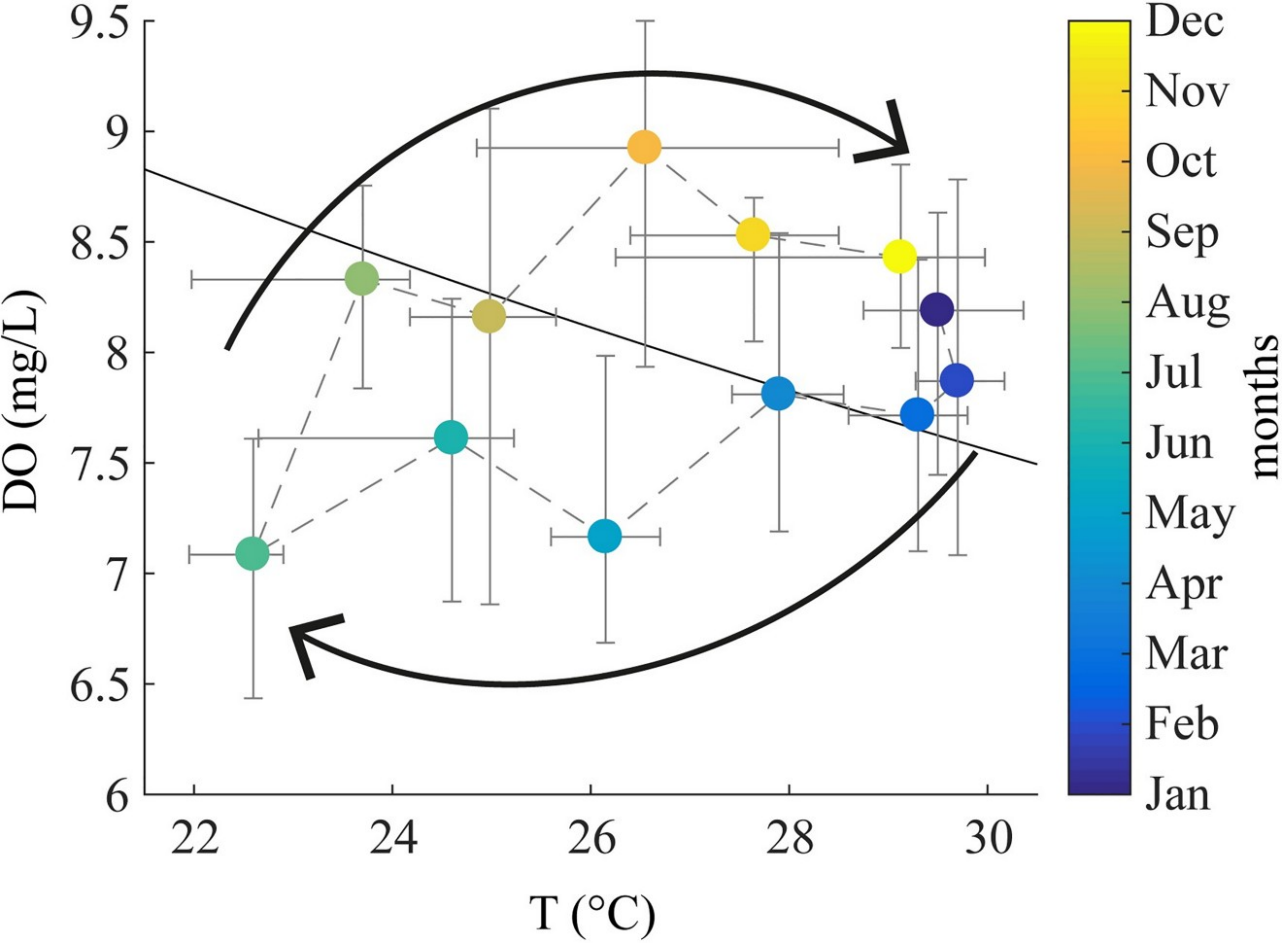
Hysteresis loop of surface water temperature and dissolved oxygen. Hysteresis loop of surface median monthly water temperature and surface median monthly dissolved oxygen. The error bars show 25^th^ and 75^th^ percentiles. All data are from the aggregated database of this study. Colours represent the months of the year and the black line represents the saturation concentration line.

In this section, we examine the relationship between surface water temperature and surface dissolved oxygen in sub-basin IV of Lake Kariba at the monthly time scale. Fig 10 shows that water temperature and dissolved oxygen concentration follow a hysteresis cycle that has to be read in the clockwise sense. Each point in the cycle represents the median value of water temperature and dissolved oxygen during the respective month. Error bars show the 25^th^ and 75^th^ percentiles. From December to February, the lake moves toward a stronger stratification phase, the surface temperature rises, and the oxygen concentration slightly decreases because of decreased solubility. From March to July the lake approaches well-mixed conditions, with not only surface temperature but also surface dissolved oxygen decreasing because of the mixing with colder and oxygen depleted hypolimnetic water. From August toward the end of the year the stratification re-starts, the well mixed layer temperature rises, and the oxygen re-equilibrates with the atmosphere. The oxygen demand in the tropical lake continues because of the high water temperature even during mixing periods, and this offsets the oxygen concentration at the surface. This analysis shows that the correlation between surface water temperature and oxygen concentrations relates to the reservoir mixing regime. In our case, the resulting hysteresis cycle supports the monomictic classification of Lake Kariba [31].

## Conclusions

By integrating satellite and in situ water temperature profiles, we quantified the spatial heterogeneity of thermal dynamics across Lake Kariba’s sub-basins. The difference between the first two riverine sub-basins and the second two lacustrine sub-basins of Lake Kariba is mainly driven by the residence time of water in each sub-basin. As a consequence, the surface water temperature in the lake increases by about 1.5°C along the longitudinal axis of the lake in both, the warm-wet and the cold-dry season. Even if the spatial variation of the surface temperature is small, its consistency can profoundly affect key physical and biological processes through nonlinear dynamics. Therefore, we believe that our analysis can support future and ongoing studies on physical and biological aspects of Lake Kariba. Moreover, the fact that the surface temperature is heterogeneous in this reservoir allows us to speculate that the different sub-basins will respond differently to climate warming, strengthening density gradients which could have cascading effects on other lake internal biogeochemical processes like photosynthesis, respiration and nutrient cycling.

Focusing on the lacustrine sub-basin IV, through a statistical analysis of the temporal database, we built frequency maps for the occurrence of temperature and oxygen conditions and estimated the AHOD for Lake Kariba to 0.8 g m^-2^ day^-1^. The frequency maps allowed us to distinguish different habitat conditions at different lake depths in the lacustrine part of Lake Kariba, and they are a promising management tool for a rapid assessment of downstream impacts of turbine outflows such as altered water temperature and dissolved oxygen concentrations. Indeed, the stratification dynamics of the sub-basins III and IV imply the potential to alter the water quality of the downstream river with consequences for the entire Middle Zambezi ecosystem. Thus, our findings suggest that future studies should focus on the last sub-basin of this lake to better understand the influence of stratification on downstream water quality. Further modelling efforts are needed to specifically assess such alterations and predict them under future possible scenarios in order to inform and guide management decisions for a more sustainable development. Developing consciousness of downstream impacts of dams may help developing strategies to mitigate such anthropogenic pressures in the context of international initiatives to reduce such impacts on the world’s great river corridors [5].

Finally, a future sustainable management of water resources in this area must be supported by ameliorating the water quality monitoring of this artificial lake. The maintenance of monitoring programmes, especially in developing countries, faces political and financial obstacles. The present study clearly illustrates the value of monitoring data for the assessment of lake-internal processes and the management of large reservoirs. For the entire Zambezi River Basin, such baseline assessments will be of high value as human activities, such as the economic development of the watershed, proceed together with climatic changes. The modern shifts of the entire river basin, in terms of climate and basin development, underscore the importance of increasing the international awareness and access to these data from Lake Kariba, as artificial reservoir construction advances together with agriculture exploitation and climate change. Predicting future responses of ecosystems to future changes relies on identifying and understanding their baseline functioning. We therefore recommend to continue the monitoring of Lake Kariba and to extend its scope to the sub-basins I to IV to improve our understanding of the riverine influence of the Zambezi. In addition, extending the measurements to the entire depth of the water column could reveal whether Lake Kariba fully mixes every year or if there are anomalous years related to its usual mixing. As Woolway & Merchant [71] recently showed, such information could be extremely important to understand if Lake Kariba is likely to switch to a different mixing regime in the future.

## Supporting information

S1 Fig Empirical cumulative distribution function and variability of lake water temperature and dissolved oxygen. Empirical cumulative distribution function of water temperature (a) and dissolved oxygen (b) at five different lake depths. All data are from the aggregated database of this study. Variability in the temperature profiles (c) and oxygen variability with depth (d). Dashed lines represent the 25th and 75th percentiles and solid black lines represent the medians of the distribution (50th percentiles).
(TIF)
