# Supplementary figures and images for: Sixty years since the creation of Lake Kariba: Thermal and oxygen dynamics in the riverine and lacustrine sub-basins
Source: PLoS One. 2019 Nov 5;14(11):e0224679. doi: 10.1371/journal.pone.0224679 (PMC6830776; doi:10.1371/journal.pone.0224679)

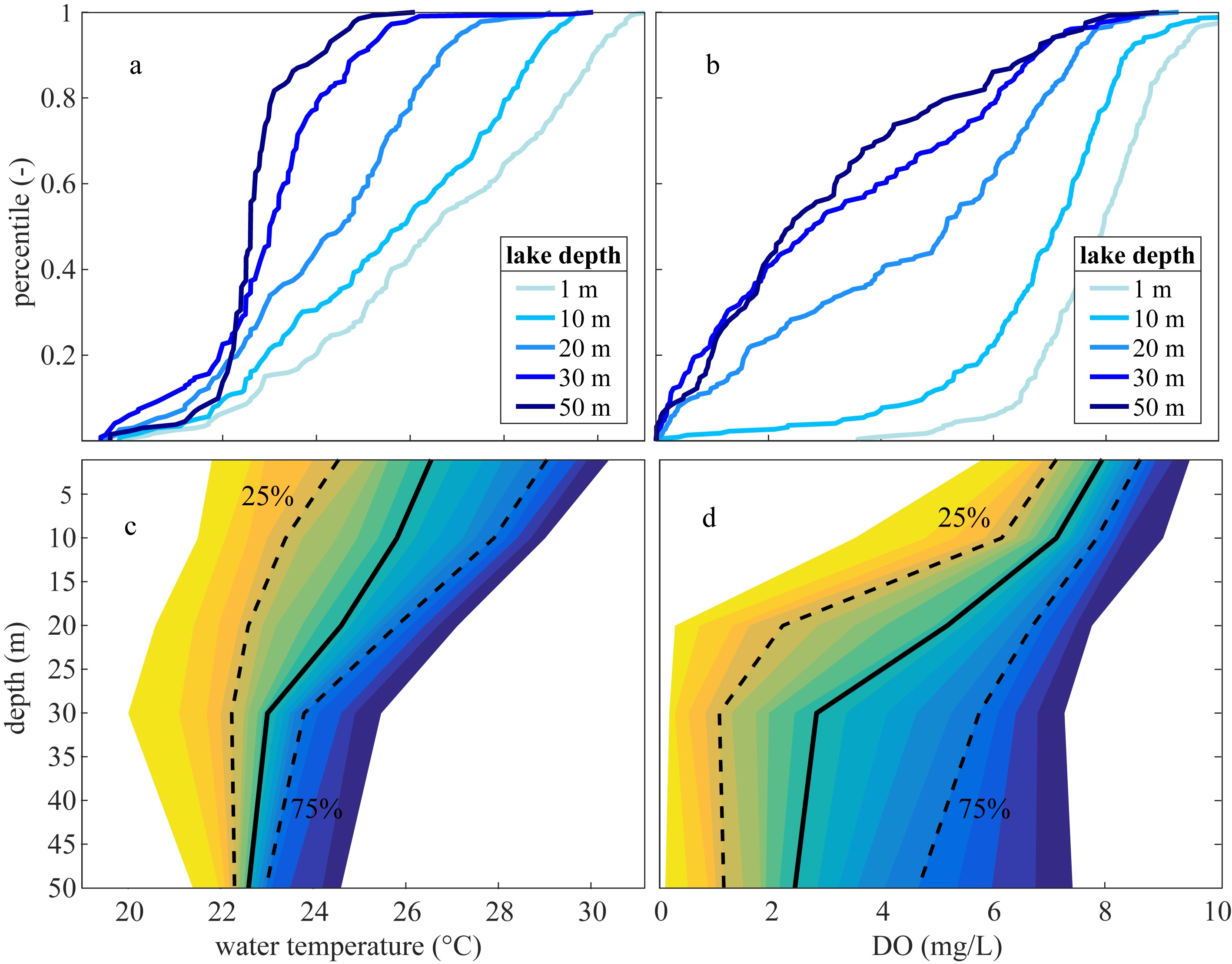

Supplement: S1 Fig — Empirical cumulative distribution function of water temperature (a) and dissolved oxygen (b) at five different lake depths. All data are from the aggregated database of this study. Variability in the temperature profiles (c) and oxygen variability with depth (d). Dashed lines represent the 25th and 75th percentiles and solid black lines represent the medians of the distribution (50th percentiles). (TIF) [file pone.0224679.s001.tif]
